# Improving treatment education in Black and South Asian women

**DOI:** 10.1007/s00520-025-10259-5

**Published:** 2025-12-22

**Authors:** Apini Patel, Luke Steventon, Jurga McLean, Shereen Nabhani-Gebara, Ayşenur Kılıç, Yogini H. Jani, Zoe Moon, Pinkie Chambers

**Affiliations:** 1 Pharmacy Department, University College London Hospitals NHS Foundation Trust, 235 Euston Road, London, NW1 2BU UK; 2 UCL School of Pharmacy, Centre for Medicines Optimisation Research and Education, University College London Hospitals NHS Foundation Trust, 235 Euston Road, London, NW1 2BU UK; 3 Royal Marsden Hospital, Fulham Road, London, SW3 6JJ UK; 4 Pharmacy Department, Faculty of Health, Science, Social Care and Education, Kingston University London, Penrhyn Road, , Kingston Upon Thames, KT12EE UK; 5 Centre for Behavioural Medicine, UCL School of Pharmacy, 29-39, Brunswick Square, , London, WC1N 1AX UK; 6 UCL School of Pharmacy, Centre for Medicines Optimisation Research and Education, University College London Hospitals NHS Foundation Trust, 29-39, Brunswick Square, , London, WC1N 1AX UK

**Keywords:** Cancer, Patient education, Ethnicity, Cultural awareness, Toxicity

## Abstract

**Purpose:**

Disparities in health outcomes for cancer patients from ethnic minority backgrounds have been reported, as consequence of direct care received or poorer access to services. As part of cancer care, patients are educated on treatments to minimise harm and maximise treatment benefit. Our evaluation was designed to understand gaps in cultural awareness of practitioners educating patients as part of their clinical practice, focussing on Black and South Asian women.

**Methods:**

This mixed methods study involved a questionnaire containing 30 questions with specific statements based on awareness, knowledge and connectivity to patients of different ethnicities, which was completed by practitioners working with patients with cancer. The questionnaire was followed by focus groups inviting patients treated with systemic anti-cancer therapy (SACT), who were >18 years of age and able to communicate for themselves. Rapid evaluation techniques were used to transcribe and analyse focus group data.

**Results:**

Ninety practitioners across the UK completed the questionnaire. Most participants were pharmacists (70%, *n* = 63). Sixty-nine percent (*n* = 62) of respondents were from the South East or London. Eighty-one percent (*n* = 73) were female. A large proportion of practitioners believed there were not enough resources available to them to adequately minority patients of different ethnicities; they identified language as a main barrier. Patients believed the complexity of language used in their education was a key area for improvement, as well as the need for more specific information on adverse events.

**Conclusion:**

Improvements should focus on better communication, adverse event management and education, representing key areas of concern identified by this study.

## Background

Cancer is amongst the highest causes of death globally [1]; however, advances in treatments, screening and diagnosis have ensured the number of deaths and mortality in the UK continue to decline [2]. Disparities in health outcomes in patients from certain ethnic backgrounds have been reported, which could be a consequence of direct care received or poorer access to services [3]. The care provided to cancer patients includes education to minimise harm from treatments and to maximise benefit.

Amongst ethnic minority groups, Black and South Asian women face the realities of racial disparities in cancer care. Black women with breast cancer from Caribbean and African backgrounds are twice as likely to receive a late-stage diagnosis than white British women in England [4]. Furthermore, they are more likely to be diagnosed with the more aggressive sub-type of triple negative breast cancer which bears poorer prognosis and means that early diagnosis of the cancer is crucial to maximise survival [5]. Similarly, women from South Asian backgrounds are more likely to receive a later-stage diagnosis of ovarian and breast cancer [6].

Inequalities in care for these women continue along the continuum of treatment. In the chemotherapy phase, where timely treatment has been proven to improve outcomes, Black women with breast cancer are more likely to encounter delays in initiation than other ethnic groups [6]. Although factors such as socio-economic status, access to healthcare, symptom awareness and cancer beliefs can impact this period, they do not fully explain the differences observed [7]. Furthermore, symptoms and adverse events (AEs) from chemotherapy are also thought to differ between different races and ethnicities [8]. For example, a high rate of AEs has been reported in Black women receiving long-term endocrine therapy following chemotherapy for endocrine receptor (ER) positive breast cancer, leading to a negative impact on adherence and outcomes [9].

Healthcare professionals play an essential role in educating and empowering patients to identify and manage AEs associated with anti-cancer treatments, and to promptly report any symptoms which may require escalation to healthcare providers. Additionally, tailored education can improve adherence to cancer treatments and directly impact their effect [10]. Highlighting good practice for healthcare professionals and the need to provide patients with high-quality information about their treatment is also crucial for patient safety [11].

It is unclear whether cancer healthcare professionals are sufficiently equipped and can provide accurate and relevant information that is tailored to patients from different backgrounds and cultures, to suit the specific needs of individuals. There are several resources that can support the development of health professionals to become culturally competent. The ACT cultural model [12] provides a set of guiding principles for culturally appropriate care, to support high-quality educational interventions for health professionals. A, C and T stand for the three core domains: Activate consciousness, Connect relations and Transform to true cultural care. Activate consciousness relates to obtaining knowledge, increasing awareness (intrapersonal, interpersonal and organisational) and heightening sensitivity. Connectivity between key stakeholders in care is considered vital and this refers to personal connectivity, community connectivity and interprofessional connectivity. Finally, to understand existing barriers and transform practice into true cultural care with structural, it is vital to ascertain the following. The major barrier to culturally competent care with awareness of structural barriers caused by social injustices being key. The domain refers to healthcare professionals adapting themselves based on understanding patterns of disadvantaged groups.

Our evaluation was designed to understand gaps in cultural awareness of practitioners who provide education to patients as part of their clinical practice, focussing on Black women and South Asian women, using the ACT model as a framework [12]. Furthermore, we describe the experiences of these women with respect to treatment and the education provided to them when initiating systemic anti-cancer treatments (SACT) and throughout their treatment course.

## Methods

We conducted a study employing a mixed methods approach to achieve our aims. This consisted of two phases: the first was a questionnaire and this was followed by patient focus groups.

### Online questionnaire

#### Questionnaire development

To evaluate practitioner practices when educating patients of different ethnicities, we developed an online questionnaire. The questionnaire was designed to collect both quantitative and qualitative data on practitioner education practices for patients from different ethnicities and cultures. The questionnaire was designed to produce informative results whilst being realistic to complete for a busy, time-limited healthcare professional. It comprised three parts and contained 30 questions in total. Part 1 captured the demographics of the practitioner; part 2 was to understand practitioner awareness and perceptions of their own knowledge regarding culture through 10 key statements requiring multiple-choice scaled responses, using a 5-point Likert scale ranging from 1, indicating ‘strongly disagree’, to 5, ‘strongly agree’. The ACT model was used to develop these statements based on awareness, knowledge and connectivity to patients. Lastly, part 3 allowed participants to free-type particular barriers and challenges in educating patients from different ethnicities. Open-ended questions were included to gain a deeper understanding of topics, and an additional option to add further comments was provided at the end of the questionnaire. The Checklist for Reporting Results of Internet E-Surveys (CHERRIES) has been adhered to in reporting results [13].

Face and content validity was affirmed via review from British Oncology Pharmacists Association (BOPA) Equality, diversity and Inclusion (EDI) and research committees and review from specialists in questionnaire design [14, 15]. Following suggested edits to wording, authors AK, YJ, SNG and JM further reviewed and piloted the questionnaire, examining questions to ensure that objectives were appropriately met.

#### Questionnaire administration

The questionnaire was open from 24th November 2023 to 15th December 2023 and was disseminated via BOPA using the Microsoft Forms® platform. BOPA was chosen as the organisation has over 500 members that are pharmacy staff in the UK. Pharmacists are a key professional group alongside chemotherapy nurses that provide patients with tailored information around oral SACT. BOPA members were directly contacted via email and the questionnaire was also disseminated through social media and was widely distributed to reach other health professionals via this route. The questionnaire was distributed via email at University College London Hospital NHS Foundation Trust (UCLH), on social media platforms and to BOPA membership. The questionnaire was distributed to staff working within the cancer division at UCLH, as findings would be used to inform local changes to practice.

### Missing data

Mandatory answering was used for various questions, both open and pre-defined, therefore posing a risk of inaccurate data. However, there was no time limit set on the survey to allow participants to complete it in the time they felt was needed to answer appropriately.

### Focus groups

To describe the experiences of Black and South Asian Women, we invited patients from two cancer charities that focussed on ethnic minority patients: The Leanne Pero Foundation (via cancer project Black Women Rising (BWR)) and the Asian Women Cancer Group (AWCG). Both groups are dedicated to supporting women with cancer with help, information and advice through their journey. Patients were invited if they had been treated with SACT, were able to communicate for themselves and were >18 years old. To describe the work, we were doing and encourage participation, a preliminary visit was made to the ACWG. A flyer was then developed and circulated via newsletters and social media for both charities. A total of 19 women responded to advertisements and agreed to participate in focus groups with knowledge that findings would be used for development of services, guide future research and be reported in publications. A total of 3 focus groups were conducted: one face-to-face with AWCG and two virtual groups with BWR, according to the availability of patients that these charities were in contact with. Patients were offered a gift voucher for £25 for their participation.

As the purpose of our work was primarily to direct service changes, we used a rapid evaluation approach [16]. A semi-structured protocol was followed, which guided questioning and included questions around patients’ experiences of the education received about cancer treatment and resources and support given. Participants were asked about cultural considerations that they believed were important when commencing SACT, as well as some barriers that Black and South Asian women may face in their treatment journeys. Lastly, they were invited to put forward their ideas for improvements that could be made to SACT services.

Each focus group was led by two facilitators: one leading the questions (AP) and the other being an experienced researcher taking field notes (PC). Both facilitators were bilingual (Gujarati) which was valuable in the face-to-face group with the AWCG. Each focus group was started with an open-ended question to encourage discussion and lasted for 90 min. Notes were taken during the focus group discussions with main points inputted into a RREAL Rapid Assessment Procedure (RAP) sheet, immediately following the groups [16].

### Data analysis

Data from the questionnaire was described using percentages, with averages calculated where appropriate. Descriptive statistics were used to analyse the data from the questionnaires using Microsoft Excel®.

The use of RREAL RAP sheets allowed for rapid qualitative methods to be used to analyse the data alongside the collection process by giving real-time findings. The data from the RREAL RAP sheets, notes and transcripts were triangulated and analysed using framework analysis to create a matrix of summarised data categories and quotations that related to the ACT cultural model.

## Results

### Questionnaire results

The questionnaire was completed by 90 practitioners across the UK. Table 1 shows that most participants were pharmacists (70%, *n* = 63), with the remaining participants being pharmacy technicians (8%, *n* = 7), doctors (10%, *n* = 9) and nurses (12%, *n* = 11). Sixty-nine percent (*n* = 62) of respondents were from the South East or London and most were female (81% *n* = 73).

**Table 1:** Overview of participants

Parameter	UCLH	National	Total (*n* = 90)
Profession			
Pharmacist	–	63 (70%)	63 (70%)
Pharmacy technician	–	7 (8%)	7 (8%)
Doctor	9 (10%)	–	9 (10%)
Nurse	11 (12%)	–	11 (12%)
Area of work			
London	19 (36%)	34 (65%)	52 (58%)
North East and Yorkshire	–	1 (1%)	1 (1%)
North West	–	8 (9%)	8 (9%)
Midlands	–	3 (3%)	3 (3%)
South East	–	10 (11%)	10 (11%)
South West	–	9 (10%)	9 (10%)
Wales	–	3 (3%)	3 (3%)
Scotland	–	4 (5%)	4 (5%)
Gender (self-identified)			
Male	4 (24%)	13 (76%)	17 (29%)
Female	16 (22%)	57 (78%)	73 (81%)
Length of time working with cancer patients			
15 years+	4 (13%)	26 (87%)	30 (33%)
10–15 years	4 (33%)	8 (67%)	12 (13%)
5–10 years	10 (42%)	14 (58%)	24 (27%)
<5 years	2 (8%)	22 (92%)	24 (27%)
Ethnicity of participant			
Black, Black British, Caribbean or African	1 (9%)	10 (91%)	11 (12%)
White	12 (24%)	37 (76%)	49 (54%)
Asian or Asian British	7 (28%)	18 (72%)	25 (28%)
Mixed or multiple ethnic groups	–	1 (1%)	1 (1%)
Any other white background	–	1 (1%)	1 (1%)
Other ethnic group	–	3 (3%)	3 (3%)

Survey responses were grouped into three areas: (1) understanding existing barriers; (2) knowledge and awareness; and (3) connectivity and communication (Table 2).

**Table 2:** Questionnaire statements and participants’ response mapped with ACT cultural framework

	% (*n*)				
	Strongly agree	Agree	Neutral	Disagree	Strongly disagree
Transform into true cultural care: understanding existing barriers					
Cultural influences and ethnicity can be linked with poor medication adherence and safety	29% (*n* = 26)	50% (*n* = 45)	14% (*n* = 13)	5% (*n* = 4)	2% (*n* = 2)
Ethnic minority groups are less likely to discuss needs or concerns about treatment	14% (*n* = 13)	48% (*n* = 43)	21% (*n* = 19)	14% (*n* = 13)	3% *n* = 2)
Ethnic minority groups are less likely to be treated with dignity and respect	6% (*n* = 5)	30% (*n* = 27)	23% (*n* = 21)	31% (*n* = 28)	10% (*n* = 9)
Knowledge and awareness					
Educational resources to educate ethnic minority group patients receiving SACT are available at my workplace	0%	14% (*n* = 13)	30% (*n* = 27)	40% (*n* = 36)	15% (*n* = 14)
At my workplace the available educational resources to educate ethnic minority group patients receiving SACT are useful	2% (*n* = 2)	9% (*n* = 8)	48% (*n* = 43)	26% (*n* = 23)	15% (*n* = 14)
I feel confident in delivering patient counselling to ethnic minority groups	4% (*n* = 4)	40% (*n* = 38)	30% (*n* = 30)	14% (*n* = 13)	5% (*n* = 5)
Connectivity and communication					
I encounter individuals from different ethnic groups when undertaking SACT counselling	36% (*n* = 32)	49% (*n* = 44)	11% (*n* = 10)	4% (*n* = 4)	0%
I always identify and remove barriers that ethnic minority patients may face in relation to their SACT	7% (*n* = 6)	31% (*n* = 28)	42% (*n* = 38)	19% (*n* = 17)	1% (*n* = 1)
I always check the preferred language of a patient before undertaking SACT counselling	17% (*n* = 15)	42% (*n* = 38)	16% (*n* = 14)	21% (*n* = 19)	4% (*n* = 4)
When I provide direct patient care, I always document assessments relevant to a patient’s ethnicity	5% (*n* = 5)	16% (*n* = 14)	36% (*n* = 32)	36% (*n* = 32)	7% (*n* = 7)

With regard to existing barriers, many respondents agreed or strongly agreed that cultural influences and ethnicity could be linked with poor medication adherence and safety (79%, *n* = 71), and that ethnic minority groups are less likely to discuss needs or concerns about treatment (62%, *n* = 56). The response to ethnic minority groups being treated with dignity and respect was more widespread.

In terms of knowledge and awareness, 55% (*n* = 50) respondents disagreed or strongly disagreed there were educational resources for ethnic minority patients available and 41% (*n* = 37) disagreed or strongly disagreed they had usefulness. There was no clear consensus around confidence in delivering education to patients from ethnic minority groups.

With regard to connectivity and communication, whilst most respondents indicated encountering individuals from different ethnic backgrounds when undertaking SACT counselling, only 21% (*n* = 19) reported document assessments relevant to the patient’s ethnicity, and 38% (*n* = 34) agreed they pro-actively addressed barriers that ethnic minority patients may face in relation to their SACT.

Participants were invited to leave free text comments, and these have been grouped manually and mapped to the domains of the framework (Table 3).

**Table 3:** Barriers to educating patients from different ethnicities

Participant comments	Participant numbers (*n* =)	ACT framework domain
Lack of resources to train healthcare professionals to be culturally competent	10	Knowledge
Lack of time to give during appointments	9	Connectivity, communication
Language barriers	19	Communication, connectivity
Lack of training specific for telephone consultations	4	Communication
No private space to conduct SACT counselling	4	Connectivity
Electronic prescribing systems not compliant for documentation or recognition of types of minority groups	3	Knowledge, connectivity
Mistrust towards healthcare professionals	4	Transform into true cultural care: understanding existing barriers, connectivity
Specific cultural barriers	11	Transform into true cultural care: understanding existing barriers
Identifying symptoms due to cultural and physical differences, for example skin changes vary as per ethnicity	2	Knowledge, awareness
Access to healthcare and not enough empowering patients to voice concerns or escalate issues if unwell	1	Awareness
Lack of individualised approach	4	Knowledge, communication

Nineteen participants perceived language to be a barrier in delivering the best care. Another common comment amongst the participants was around the lack of training and time that is allocated for patients.

### Focus group results

A total of 15 patients were included in focus groups, from the initial 19 that had expressed an interest to participate. Thirteen (89%) were patients with breast cancer and two (11%) were patients with colorectal cancer. All participants could speak English and self-identified as a woman of Black or South Asian heritage. Six participants were recruited through AWCG and 9 participants through BWR.

The following themes were identified.

#### Connectivity

Connectivity in the ACT model is important to provide culturally competent healthcare. This connectivity was a key gap with many patients. Patients described delays in diagnosis of breast cancer, which they believed to be related to cultural differences such as ‘*not being taken seriously at the GP*’ [Participant 1, South Asian, AWCG focus group] and not knowing that ‘*Black women tend to have more dense breasts*’ [Participant 7, Black, BWR focus group]; therefore, mammograms may not pick up a diagnosis. These events contributed to mistrust towards healthcare professionals, compromising the approachability of these staff by patients and raising questions about the appropriateness of care offered. Participants described the stigmas associated with being a Black woman; ‘*it happens to us as well in that Black women are expected to be more tough, and they just get on with it. So, there’s often a lack of empathy*’ [Participant 10, Black, BWR focus group].

A common theme from all three focus groups was the availability or lack of availability of psychosocial support before, during and after completing SACT. In the face-to-face focus group with South Asian women, the participants discussed how they could not tell family members of their diagnosis until their treatment was over. Reasons being that they did not want to burden their families, or they wanted to keep treatment private from the wider community. They felt that without the support of their families, groups and charities were especially important but would have liked to be offered some support at their hospitals.

#### Communication

Participants felt a lack of empathy from some practitioners. Two participants were referred to the respective charity from their local hospital; however, the remaining 13 participants had to self-advocate and seek the appropriate charity or support groups for themselves.

Difficult medical terminology and the resultant lack of understanding during consultations with healthcare professionals was discussed as a perceived failure of the health system, with a lack of initial communication resulting in patients being unaware of the potential side effects when they consented to treatment. ‘*One side effect was cancer, and they wanted me to sign to that and another was death*’ [Participant 3, South Asian, AWCG focus group].

Five participants described AEs being listed to them that were non-specific.

#### Activate consciousness

Within the ACT model, awareness and knowledge are key within the domain of activating consciousness. However, in all three focus groups, women described side effects specific to women of darker skin tones that health professionals seemed unaware of. An example was skin discolouration, which was not mentioned during SACT education.

‘*It’s a bit of a scary one. You just think what is happening like, you know, suddenly my skin’s changing this colour*’. [Participant 12, South Asian, BWR focus group]

‘*it’s only when I raised it with my oncologist that he said, Oh yes, that does happen to some patients of colour. It’s temporary, you know*’. [Participant 14, South Asian, BWR focus group]

One participant had a positive experience having specific hair-related advice given by her chemotherapy nurse.

‘*At the time I had extensions, and she looked at me, said you might want to take them out because the weight of them will pull your hair. Now that really shocked me. The lady was Portuguese and just for her to have that awareness*’. [Participant 10, Black, BWR focus group]

With endocrine therapy, six breast cancer patients across the focus groups described having significant AEs with minimal resolution or assistance from a healthcare professional, implying that they lacked the knowledge to improve the care provided.

‘*The most difficult treatment for me has been the maintenance treatment I’m on now. It was worse than all the other treatments put together so letrozole I didn’t expect to be as brutal*’. [Participant 9, Black, BWR focus group]

There was a clear lack of support and resources provided regarding endocrine therapy. One participant was made to feel she was lucky to have this treatment only as intravenous chemotherapy was not indicated. Several patients described having adherence concerns and a lack of understanding of the benefits of taking this endocrine therapy.

At the end of the focus groups, all participants highlighted improved education specific to endocrine therapy as a change that would have most impact. Tailored and specific education of all SACT was needed rather than lists and large booklets with lots of writing. A simple, large font crib sheet at the start of treatments with simple explanation was what patients wanted, but sadly none received.

Educational advice regarding diet and lifestyle was also missing and participants felt that information given was too broad and applied to those following a white Mediterranean diet, rather than rich diets of other cultures.

## Discussion

Our evaluation was developed to identify ways to improve the education for Black and South Asian Women being treated with SACT. Our work uncovered two key areas for improvement: personal connectivity and knowledge. Both the connectivity with patients and knowledge acquisition of information that was relevant to these women would mean that practitioners could tailor the education given to patients in a way that is personalised and comprehendible to them.

We found there were differences in the barriers that practitioners perceived to improving care compared to patients. Interestingly, our questionnaire revealed that practitioners perceived non-English language as a key barrier in educating those of different ethnicities, whereas patients described the complexity of the language and use of unfamiliar medical terminology as a key barrier. This disconnect may be due to the non-inclusion of non-English speakers to focus groups, but also underlines the need for better connectivity of practitioners and patients when discussing cancer treatments.

Organisational barriers were evident in the questionnaire responses, with over half of the practitioners indicating a lack of available educational resources to educate patients of ethnic minority. A solution to this could be the promotion of shared resources amongst hospitals. Other organisational issues such as limited time allocated for education was reported by many and therefore personalising patient care may be prohibited.

One of our key findings from the patient focus groups was the lack of knowledge around AEs in women of different skin tones. These AEs that were important to the women in our focus groups was not part of the education given to them. There is lack of evidence on AEs in South Asian and Black women; however, studies suggest particular some AEs such as peripheral neuropathy are more prevalent [8, 17, 18]. Skin discolouration or hyperpigmentation has been observed in the literature associated with SACT; however, this is not yet widely reported in Black and South Asian women [19, 20]. Findings from the focus groups highlighted the anxiety and distress associated with skin discolouration, coupled with lack of information as to when this would appear, cease or improve. Participants felt underinformed, with skin discolouration often recognised by the treating healthcare professional as an insignificant AE, despite the visual impact and difficulty to conceal. This particularly influenced participants that did not disclose their diagnosis to friends and family, demonstrating the taboo surrounding cancer prevalent in some ethnic minority groups [21]. Clinical trial recruitment also plays a notable role in the study of this, due to poor ethnic representation in population samples and thus lack of ethnicity-specific information available to educate patients [22–24].

Lack of knowledge about endocrine therapy in breast cancer treatment was found to affect women. This resulted in several participants finding it the most brutal part of the treatment regimen regarding toxicity. Although this is somewhat documented in the literature, support available in the UK for patients remains inadequate and may be due to the poor connectivity of healthcare professionals and patients when providing care. This lack of connectivity between patient and health professional may explain poorer adherence in Black patients to treatments compared to white counterparts [25–28].

Our study was unique in bringing together findings from a health professional survey and focus groups. There are however limitations, as our work was designed to guide service improvements, and as pharmacists are heavily involved in the education of patients, we focussed our questionnaire primarily in this group. Whilst granular details of ethnicities were not recorded, all participants identified as either Black or South Asian and could understand and speak English, therefore views about language barriers cannot be directly correlated. Additionally, two focus groups were completed virtually to allow for the inclusion of patients who were only able to participate without physically travelling to our site. However, a key strength was that the three focus groups were cohesive and included participants from different areas of the UK. The mixed method approach we used highlighted that culture plays a role in healthcare and patient experiences, reflecting other studies in ethnic minority patients [18, 29].

This work explored the awareness of cultures and how these impacts understanding when discussing SACT with patients. Implementing the themes identified in this study into practitioner training is crucial to achieve cultural awareness and encourage reflection of personal biases. Furthermore, fostering organisations to share methods and resources currently in use can help to break barriers in care. Further research into AEs of SACT and their management should be a priority to better inform patients. Data from the focus groups implied that education around efficacy of treatment, side effects and improved communication with practitioners are tangible methods of improving care in this cohort. Other research into SACT-associated AEs in ethnic minority patient groups is an area of unmet need. Ensuring clinical trial recruitment is diverse would produce results that are generalisable to all ethnicities.

## Conclusion

Understanding that a one-size-fits-all approach is outdated and insufficient for today’s cancer patient population is vital to improve patient education. Individualised patient care and adapting to patient beliefs and concerns in relation to their culture is expected to improve treatment adherence and outcomes for WOC. Interventions should focus on barriers regarding communication, psychosocial behaviour, adverse event management and education, representing key areas of concern identified through this study.

## Data Availability

No datasets were generated or analysed during the current study.
